# Deployment and Verifications of the Spatial Filtering of Data Measured by Field Harvesters and Methods of Their Interpolation: Czech Cereal Fields between 2014 and 2018

**DOI:** 10.3390/s19224879

**Published:** 2019-11-08

**Authors:** Tomáš Řezník, Tomáš Pavelka, Lukáš Herman, Šimon Leitgeb, Vojtěch Lukas, Petr Širůček

**Affiliations:** 1 Department of Geography, Faculty of Science, Masaryk University, Kotlářská 2, 611 37 Brno, Czech Republic; 409021@mail.muni.cz (T.P.); herman.lu@mail.muni.cz (L.H.); leitgeb@mail.muni.cz (Š.L.); 2 Department of Agrosystems and Bioclimatology, Faculty of Agronomy, Mendel University, Zemědělská 1, 613 00 Brno, Czech Republic; vojtech.lukas@mendelu.cz (V.L.); qqsiruce@node.mendelu.cz (P.Š.)

**Keywords:** field harvester, yield mapping, sensor measurements, interpolation, data filtering

## Abstract

Yield mapping is a subject of research in (precision) agriculture and one of the primary concerns for farmers as it forms the basis of their income and has implications for subsidies and taxes. The presented approach involves deployment of field harvesters equipped with sensors that provide more detailed and spatially localized values than merely a sum of yields for the whole plot. The measurements from such sensors need to be filtered and subject to further processing, including interpolation, to facilitate follow-up interpretation. This paper aims to identify the relative differences between interpolations from (1) (field) measured data, (2) measured data that were globally filtered, and (3) measured data that were globally and locally filtered. All the measured data were obtained at a fully operational farm and are considered to represent a natural experiment. The revealed spatial patterns and recommendations regarding global and local filtering methods are presented at the end of the paper. Time investments into filtering techniques are also taken into account.

## 1. Introduction

Precision agriculture is defined as “management strategy that gathers, processes and analyzes temporal, spatial and individual data and combines it with other information to support management decisions according to estimated variability for improved resource use efficiency, productivity, quality, profitability and sustainability of agricultural production” [1]. The main goals of (precision) agriculture are devoted to the maximization of economic profit on the one hand and the minimization of negative impacts on the environment on the other. The most tangible negative consequences of agricultural activities can be seen in the degradation of soil (e.g., erosion and the loss of organic content and/or biodiversity) and the pollution of (ground) waters by residues from fertilizers [2] as well as pesticides. In all such cases, precise spatial information is the key to the most efficient as well as sustainable usage of arable land 

Conventional farming assumes that each field is a homogeneous area. For example, the same amount of fertilizer/pesticide is applied to each area of the plot. As stated by Aurenhammer [3], the concept of precision farming adds, among other things, the perspective of variable rate treatment. Precision farming techniques count and rely on the heterogeneity of a plot, defined in terms of so-called yield productivity zones (also known as management zones) that reveal areas with lower, average, and higher yields. The identification of yield productivity zones is the key to becoming more efficient from both the economic as well as ecological points of view. As such, the identification of yield productivity zones has been discussed for the last few decades (van Wart et al. [4], van Ittersum et al. [5] and Chen et al. [6]). Meanwhile, universal indirect methods are also being developed for the assessment of actual crop growth and yield on the basis of remote sensing (see e.g., Azzari [7], Bauer [8], Doraiswamy et al. [9], Lobell [10], Mirvakhabova et al. [11], Mulla [12] or Novoa et al. [13]). 

Credible detailed data on crop yield can be used to determine variable rate treatments. Data from field harvesters represent the most detailed as well as the most credible source of yield information. In spite of this, field harvesters provide measurements with bias. Bias in datasets created by field harvesters corrupt the results, meaning that soil cannot be cultivated correctly. As suggested by Blackmore et al. [14] as well as Arslan et al. [15], such errors might arise for the following reasons: for example, the occurrence of unexpected events during the harvesting process, leading to unusual behavior on the part of the machine; the trajectory of the field harvester; errors caused by the wrong calibration of the yield monitor and presence of animals, adverse weather conditions, etc.

Measured yield data need to be filtered to reduce these biases, as documented by Gozdowski et al. [16], Spekken et al. [17], Sun et al. [18], Lyle et al. [19], or Leroux et al. [20]. These papers analyze the source of bias or errors during yield mapping [14,15,19] or propose various filtering methods or methodologies [16,17,18,20]. However, none of these papers attempts a comparison using statistical methods to identify the differences between various methods of yield data filtering. 

**Figure 1:**
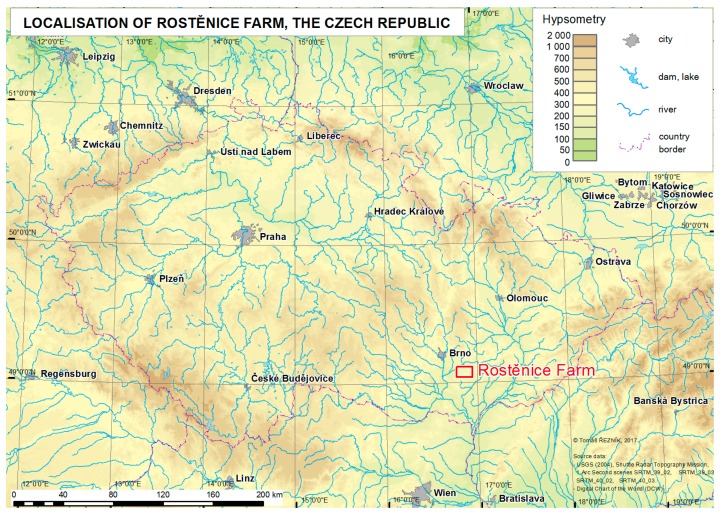
Geographical location of the Rostěnice Farm.

Consequently, this paper focuses on a statistical analysis and filtering of data measured by field harvesters at the Rostěnice Farm in the Czech Republic (see Figure 1 and/or Figure S1, as well as Figure S2 in Supplementary for detailed information) between the years 2014 and 2018. Our research deals with filtering of farm machinery sensor measurements with regard to the yield of cereals. The research in this paper deals with certain types of field sensor data of cereals at a fully operational farm; more details are provided in Table 1. 

The main goal of this paper is a relative comparison of interpolations from

1. (Field) measured data;
2. Measured data that were globally filtered; and
3. Measured data that were globally and locally filtered.

So far, relative differences between the three interpolation approaches have not been analyzed in any previous research and remain open as scientific questions.

We seek answers to the following scientific questions (research objectives):

1. What are the relative differences between interpolations from data measured by a field harvester and interpolations from identical data that are processed by global filters?
2. What are the relative differences between interpolations from data measured by a field harvester and interpolations from identical data that are processed by global and local filters?

Our research, as presented in this paper, aims at statistical (spatial pattern) analyses of interpolations from data measured in a field on the one hand and filtered data (globally/globally and locally) on the other hand. Information on relative differences is required by both the agronomical practice as well as raised in the analyzed scientific papers as follow-up activities. The follow-up applied research is beyond the scope of this paper due to the extent and time constraints. Time investments into filtering techniques presented in the Discussion Section are the only exception.

## 2. Materials and Methods

### 2.1. Study Site

Data measured by a cereal field harvester were used to analyze and evaluate the approaches of spatial filtering and interpolation. Data acquisition was conducted at the Rostěnice cooperative farm in the south-eastern part of the Czech Republic (see also Figure 1). The farm, Rostěnice a.s. (N49.105 E16.882), manages over 8300 ha of arable land in the South Moravian region of the Czech Republic. The average annual rainfall is 544 mm and the average annual temperature is 8.8 °C. Within the managed land, the most prevalent soil types are Chernozem, Cambisol, haplic Luvisol, Fluvisol near to water bodies, and, occasionally, also Calcic Leptosols. The main programme is plant production, where the main focus is on the cultivation of malting barley (2500 ha), maize for grain and biogas production (2500 ha), winter wheat (2000 ha), oilseed rape (1000 ha), and other crops and products such as soybean and lamb. The average production intensity is 6 t/ha for malting barley, 7 t/ha for winter wheat, 10 t/ha for grain maize, and 4 t/ha for oilseed rape.

The fields are located in sloping terrain; for this reason, conservation practices with respect to soil tillage have been implemented to reduce soil water erosion. The farm has applied long-term soilless cultivation (mostly choppers) on its land, leaving all straw after harvest on the land. The high spatial variability of soil conditions in the southern part of the farm has led to the adoption of precision farming practices, such as the variable application of fertilizers (since 2006) and crop yield mapping by field harvesters (since 2010).

### 2.2. Sensor Measurements

Data were measured for two fields by a CASE IH AXIAL FLOW 9120 field harvester equipped with an AFS Pro 700 monitoring unit over three years: from 2014 to 2017 for the Pivovárka field and from 2016 to 2018 for the Přední prostřední field. Note that measurements for the Pivovárka field and year 2015 were not available due to failure of the monitoring unit. The measurements were of GNSS-RTK quality (Global Navigation System of Systems—the Real Time Kinematics), i.e., they provided a spatial resolution of less than 0.1 m (see e.g., Jedlička et al. [21]). Measurements were taken continuously each second at an average speed of 1.55 m·s^–1^, recommended as optimal at the Rostěnice Farm for cereal harvesting by the CASE IH AXIAL FLOW 9120 field harvester. The harvesting width was 9.15 m. The AFS Pro 700 monitoring unit was calibrated at 15 randomly selected locations to produce yield maps that were as precise as possible. Calibration of the AFS Pro 700 monitoring unit was based on explicitly measured weight of the field harvester’s container and harvested acreage. The used CASE IH AXIAL FLOW 9120 field harvester could not measure the weight by itself, weight measurements were therefore external. Such calibration was an iterative process, repeated three times as a standardized action of the Rostěnice farm. The monitoring of yield was conducted as on-the-go mapping by recording grain flow and moisture continuously over the whole plot area. The spatial distribution of the measured yield values at Rostěnice Farm is shown in Table 1. The crop type did not influence the spatial density of the performed measurements.

**Figure 2:**
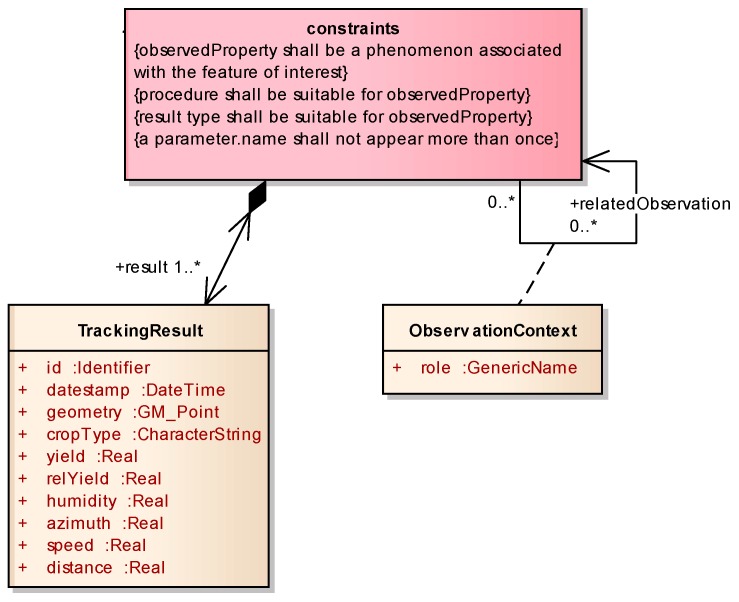
The UML (Unified Modelling Language) class diagram of the data model used for measurements. The data model represents a conceptual specialization of ISO 19156 (not depicted fully due to its complexity; focusing only on the specialised class “TrackingResult”).

The measurements were stored directly in the field harvester and manually copied to a USB flash drive after the end of operations on the pilot fields. The conceptual schema for measurements by a field harvester follows ISO 19156 Geographic information—Observations and Measurements [22]. The results of measurements comprise the locations and attributes described in the UML (Unified Modelling Language) class diagram in Figure 2. The used data model represents a specialization of the Open data model for precision agriculture, as defined by Řezník et al. [23]. Figure 2 depicts the ISO 19156 schema only partially; the class “TrackingResult” demonstrates the specialization of the ISO 19156 generic abstract class “Result”.

### 2.3. Filtering of Yield Data

**Figure 3:**
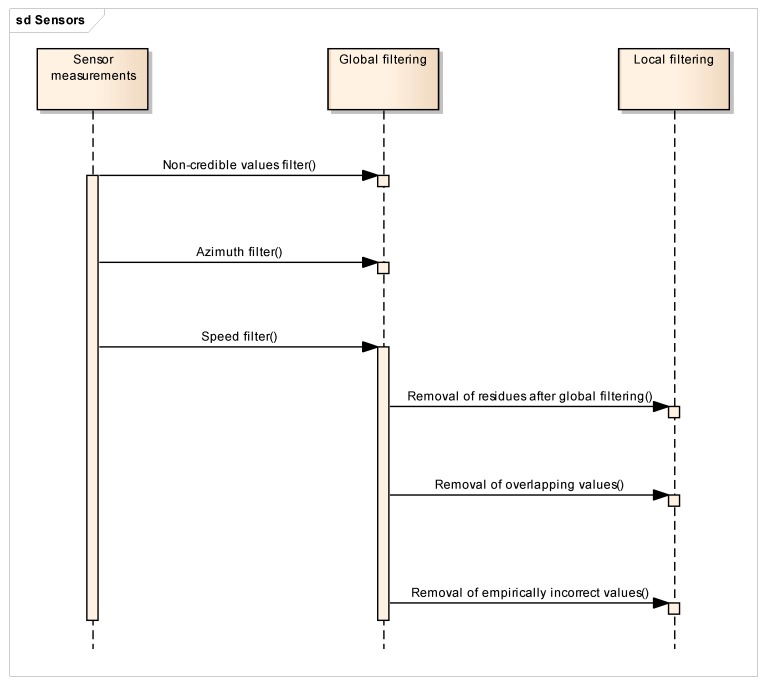
The overview UML sequential diagram of global and local filtration methods.

Our approach regarding the filtering of measured data can be divided into two parts—the cleaning of global and local outliers as follows:

(a) The cleaning of global outliers (hereinafter global filtering) removes non-credible values within the whole dataset by means of the statistical analysis of attribute values, namely yield measurement, speed of the vehicle, or direction of vehicle movement (see also the UML sequence diagram in Figure 3).
(b) The cleaning of local outliers (hereinafter local filtering) focuses afterwards on particular parts of the dataset in a higher level of detail, and is mostly based on the neighborhood of data point values or pattern recognition.

Details of both global and local filtering are described in greater detail below, as well as in the results section. A general processing sequence is depicted in Figure 3.

Global filtering uses statistical, i.e., non-spatial, analyses and methods for detecting non-credible (yield) values. Non-credible in this sense means that there is a high probability that (1) a value in a given location was measured incorrectly, or (2) a value was not achievable within the measurement process, or (3) the conditions of measurement do not seem credible.

Three global filters were used for detecting incorrect outliers:

the range of realistic yield values;
the direction of harvesting;
the speed of a field harvester.

The range of realistic yield values follows the thresholds established by the structure of the data, both empirically and on the basis of the frequency curve (see Equation (1)).

(1) $$v_{s}\in \mu \pm 2\sigma ,$$

where:

*v_s_*: harvester velocity,
*μ*: mean velocity,
*σ*: standard deviation of velocity.

The statistical file follows normal distribution. The measured data were considered as significant within the interval of two times the standard deviation, i.e., 95.5% of all values. Such a value was also discussed with, and approved by, the agronomists at the Rostěnice Farm. In other words, points outside the threshold values of 50%, and 150% for relative yield were considered nonrealistic and were excluded from the dataset (the average value of the relative yield for the whole dataset was 100%). Such interval also corresponds to ± 2 standard deviations. We accepted the removal of some realistic values as a lower threat in comparison to keep keeping non-realistic values in a dataset intended for interpolation; this view is based on the assumption of agronomists from the Rostěnice farm as well as exploratory statistical analyses of measured fields.

The second global filter selected the points that were harvested in a direction different to the dominant direction(s). Such selection applies mainly to peripheral areas of fields, so-called headlands, that are from a trajectory point of view cultivated in a different way to the rest of the field.

The third global filter was based on the speed of a field harvester. The speed of a field harvester should be as constant as possible to ensure data are valid. The amount of measured yield is different if a field harvester passes with a considerably lower or higher speed. A harvesting speed of 1.55 m·s^−1^ was the mean value for speed measurements (as also indicated in Section 2.2). Harvesting speed influences yield measurements. The lag time between harvesting in a certain position and sensor measurement was equal to 12 s. The locations of measurements can be degraded in cases where a field harvester is considerably faster or slower than the mean speed. Values excluded by the global speed limitation filter were those that exceeded the limit of three times the standard deviation, i.e., 0.3% of the measured values.

Local filtering focuses on data in greater detail and is based on differences between neighboring cells and/or patterns. A decision on the credibility of analyzed data needed to be made also in this case. General algorithms with various flexibilities have been developed for local filtering [18,19]. Such universality comes at a price, however, as none of the developed algorithms can provide sufficient results in all analyzed cases, i.e., for all years and for all fields. Basically, the more general the algorithm is, the wider its usage, but the results are less precise. Local filtering brings the most precise results with regard to domain knowledge, e.g., measurements, the processing of data, and the history of the yield, the knowledge of the data, of the situation, and of the problematics in general. The development of other general local filtering algorithms was beyond the scope of this paper due to the time and effort such a task would require. Local filtering comprises a set of subjective methods. In general, points are excluded manually.

### 2.4. Applied Interpolation Methods

Interpolation methods were used to compare, verify, and evaluate the differences between interpolated surfaces derived from field sensor measurements that were (1) obtained directly from the field harvester, (2) processed by global filters, and (3) processed by global and local filters. The preconditions for the Simple Kriging method were met [24,25]. When following Cressie [24], it is assumed that we know µ exactly, and also that data locations are known. It was confirmed that the data of measured values had normal distributions, were homogeneous, and did not show any significant trends. The parameters of the interpolations of each model were computed by means of Exploratory Spatial Data Analysis (also known as ESDA) in ArcGIS 10.6 software. 

### 2.5. Verification Methods

Map algebra was used for the spatial comparison of measurements that were (1) unfiltered, (2) filtered globally, and (3) filtered globally as well as locally. The relative differences were used as defined in Equation (2). Relative differences are often used as a quantitative indicator of quality assurance and quality control for repeated measurements where the outcomes are expected to be the same.

(2) $$dv=\frac{v_{a}-v_{b}}{v_{a}}\times 100,$$

where:

*d_v_*: relative value difference (%),
*v_a_*: unfiltered interpolated yield value,
*v_b_*: filtered interpolated yield value.

Statistical comparison required several transformations. First, interpolated raster data (the comparison of data measured by a field harvester, data that were processed by global filters, and data that were processed by global and local filters) needed to be converted to discrete points. These points were distributed regularly, and their distances corresponded to the pixel sizes of the input raster (5 m). In addition, the attribute values of these points were statistically analyzed. Due to the frequency of points and the size of the studied fields, it was expected that attribute values would have a normal distribution. Student’s *t*-test [26], specifically the dependent two-sample *t*-test, was chosen as the appropriate statistical test.

## 3. Results

### 3.1. Global Filtering

**Figure 4:**
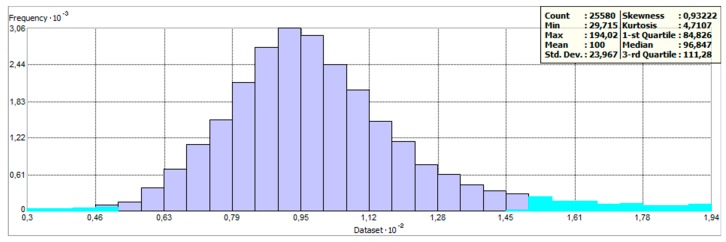
Distribution of measured yield values at Přední prostřední field in 2017 (excluded values highlighted by light blue color).

**Figure 5:**
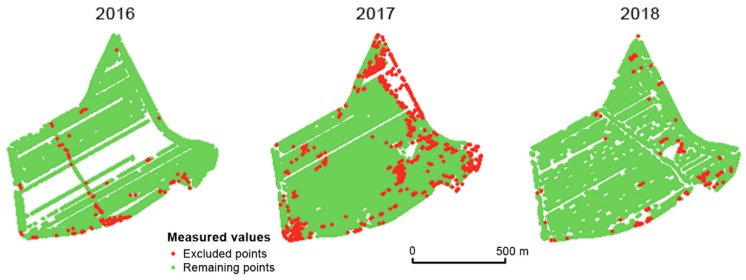
Excluded points with non-credible values of very high and very low relative yield in the field Přední prostřední. The identical global filter was used.

Thresholds for the range of yield values were defined as described by Equation (1). Figure 4 depicts the distribution of values as a histogram. It is assumed that values lower than 50% and higher than 150% of relative yield were the result of errors that occurred during the measurement process. Another source of this kind of error might be unexpected events or conditions on the field—for example, missing crops on part of the field (because of animals, weather, etc.). Examples of excluded points as well as comparisons between years can be seen in Figure 5.

**Figure 6:**
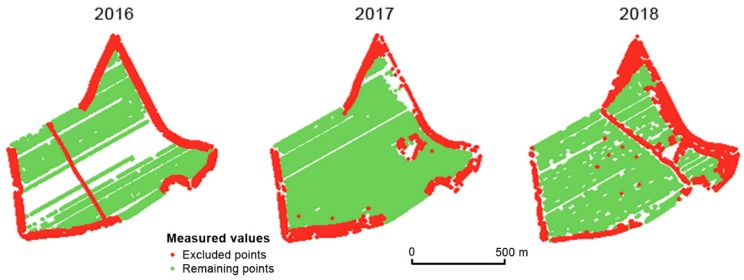
Points excluded by the dominant direction global filter in the Přední prostřední field.

The second global filter aims at the direction of harvesting as described in Section 2.3. In general, farmers do not consider headland areas as (1) yield prosperous areas or as (2) representative sensor measurement areas. There are mostly ecological reasons for the different harvesting directions applied to headland areas. The global filter on direction also excluded the turns the field harvester had to make between areas. Considering directions different to the dominant direction(s) within the main area of the field, these data did not seem credible either. Two major errors were observed. Firstly, when the field harvester was not moving in a straight line, the cutting bar did not collect the crop regularly because the angle of ‘attack’ was changing. Secondly, data from locations that were harvested in a direction different to the dominant direction were non-credible. According to Figure 6, it seems that the Přední prostřední field was divided by a temporary road in 2018 (this was confirmed by the agronomists at the Rostěnice Farm).

The prevailing direction of harvesting needed to be set specifically for each field, and it was defined through an azimuth. In some cases, however, the field harvester did not follow straight lines but curves. We identified several influencing factors, of which the terrain was the most important. The prevailing azimuth values for both fields were set from 50° to 70° in one direction and from 230° to 250° in the reverse direction (see Equation (3) and Figure 6). Measurements beyond the azimuth thresholds were removed.

(3) α∈[50,70]°∪[230,250]°,

where:

α: azimuth of harvester trajectory,
[50,70]°, [230,250]°: ranges of valid azimuth values.

**Figure 7:**
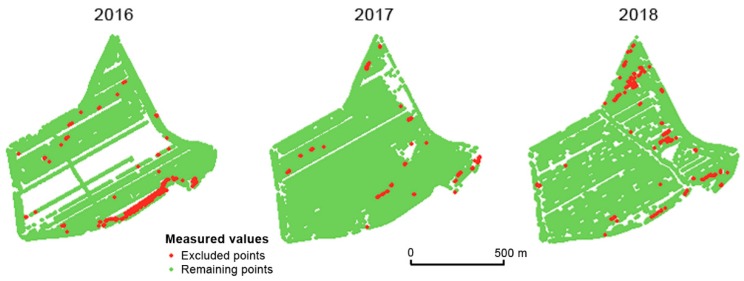
Points excluded by the global speed limitation filter in the Přední prostřední field.

The third global filter aims at the speed of a field harvester. Areas in which the speed of the field harvester was very low correlated with higher measured values of yield and vice versa. According to agronomists at the Rostěnice farm, the speed of the field harvester changed for the following reasons: obstacles in the field, mechanical issues with the field harvester, or the behavior or inattention of the driver. Areas excluded by the global speed limitation filter are depicted in Figure 7.

**Figure 8:**
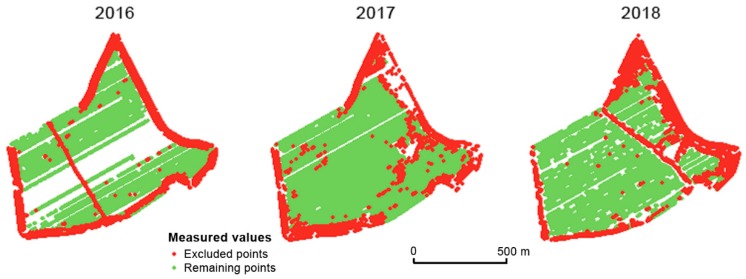
Points excluded after applying all three global filters (extremely low/high relative yield, dominant direction, and speed limitation) in the Přední prostřední field.

Figure 8 shows the measurements excluded by three global filters. Two conclusions may be reached. Firstly, the excluded measurements appear mainly in headland areas. Secondly, the excluded measurements vary considerably over the years (see Figure 8). It is assumed that the points excluded by means of global filtering are errors made during the harvesting process as well as during the measurements themselves. Any spatial correlation between excluded measurements across years was not proven.

### 3.2. Local Filtering

**Figure 9:**
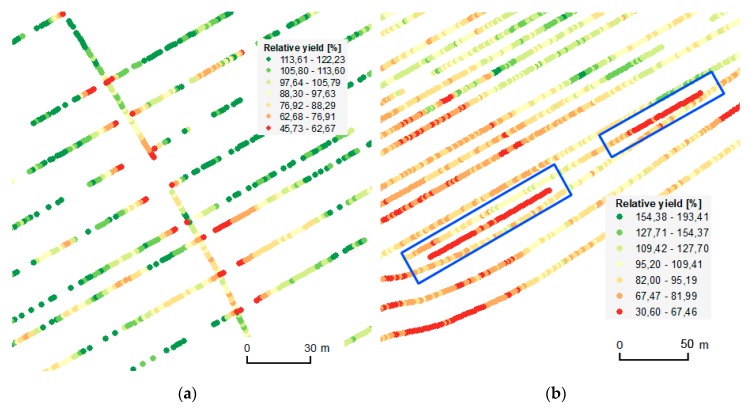
Examples of overlapping (**a**) and partially overlapping (**b**) trajectories. The single-oriented trajectory was the first, while all the remaining trajectories depict the subsequent movements of the field harvester. Measured data were divided into classes according to the Jenks Natural Breaks method [25].

Several issues were considered with respect to local filtering. First, overlapping measurements from the positional point of view were removed. In such cases, identical areas were “harvested” and therefore logged twice in the measured data. Values close to zero were measured during the second pass as almost the whole yield was harvested in the first pass. An example of an overlapping trajectory is depicted in Figure 9a. Local filtering methods analyze those areas again and remove leftover erroneous measurements.

Figure 9a represents the most obvious case of overlapping trajectories. In many other cases, trajectories were not crossed explicitly. That is, trajectories were overlapped only by a portion of the cutting bar, as depicted in Figure 9b. In such cases, the partial use of the cutting bar leads to non-representative values.

The cutting bar of the field harvester in the conducted pilots was 9.15 m wide. The values of any points in neighboring rows lying within the adjacent path of the cutting bar are misleading and can therefore be considered as errors. As confirmed by the Rostěnice agronomists, overlaps of this kind usually occur because crop leftovers must be harvested.

**Figure 10:**
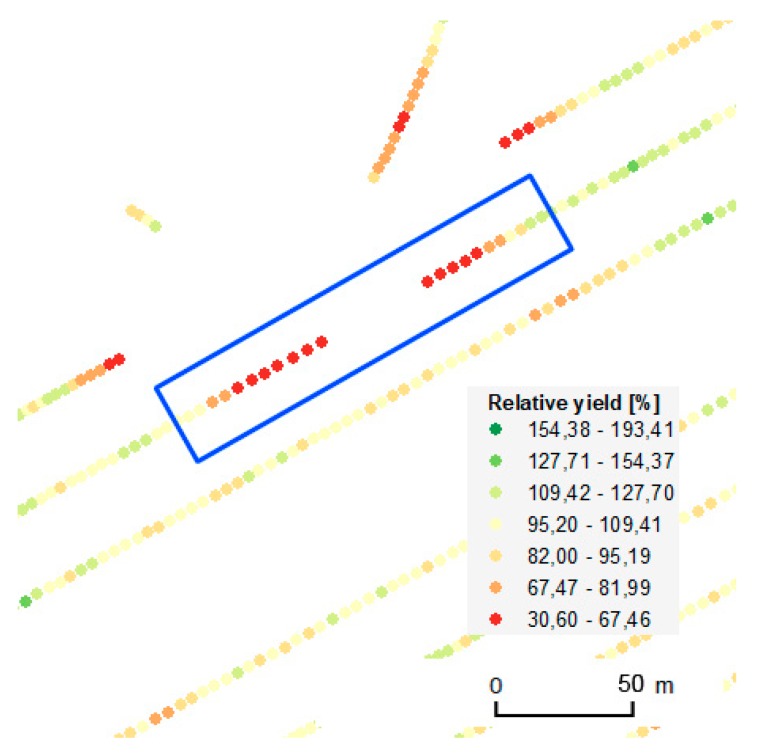
Example of gaps in measurements within a row.

The third applied local filter targeted the gaps in measurements within rows; see also Figure 10. In many observed cases, gaps within rows were connected with a decrease in the measured yield. Sudden decreases occurred for example when the field harvester had to raise its cutting bar in order to avoid an obstacle.

Erroneous measurements with sudden decreases in values were also observed at the ends and beginnings of gaps in measurements within a row. Such patterns were observed when a part of the crop was missing because of weather (strong rain and hail, drought, etc.), animals, or other unexpected events. The causes of such gaps—and therefore the respective decreases in measured yield—were often unique to the particular harvest or year, as no correlation was identified across years. The majority of such erroneous measurements were excluded by global filtering. Values of neighboring rows were also taken into account to distinguish between errors and coincidences when gaps correlated with zones with lower yield.

**Figure 11:**
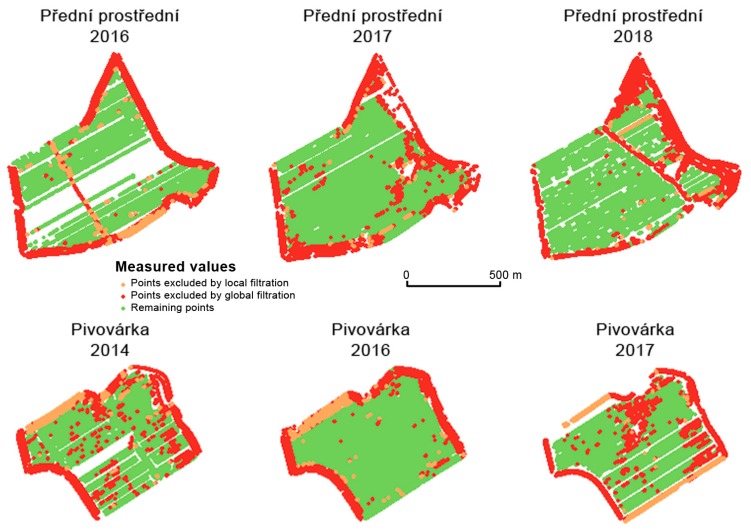
Spatial distribution of measurements filtered by means of global and local filters.

Figure 11 together with Table 2 and Table 3 depict the spatial distribution as well as the absolute amounts of measured data that were filtered by means of global as well as local filters. A considerable number of measurements were excluded in the case of the Přední prostřední field in 2018 because of headland areas and because of the high number of gaps inside the field. The year 2018 was extremely dry in this part of the Czech Republic, which had an impact on the yield and on the number of gaps.

### 3.3. Interpolation of Yield Data

**Figure 12:**
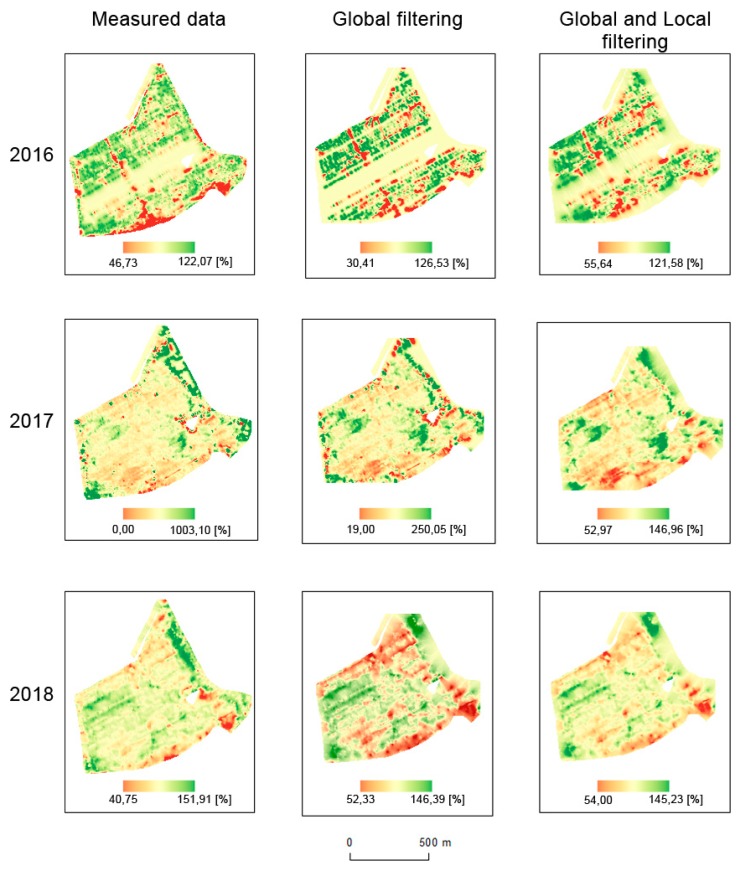
Interpolations of measured, globally filtered, and both globally and locally filtered data for the Přední prostřední field (%).

**Figure 13:**
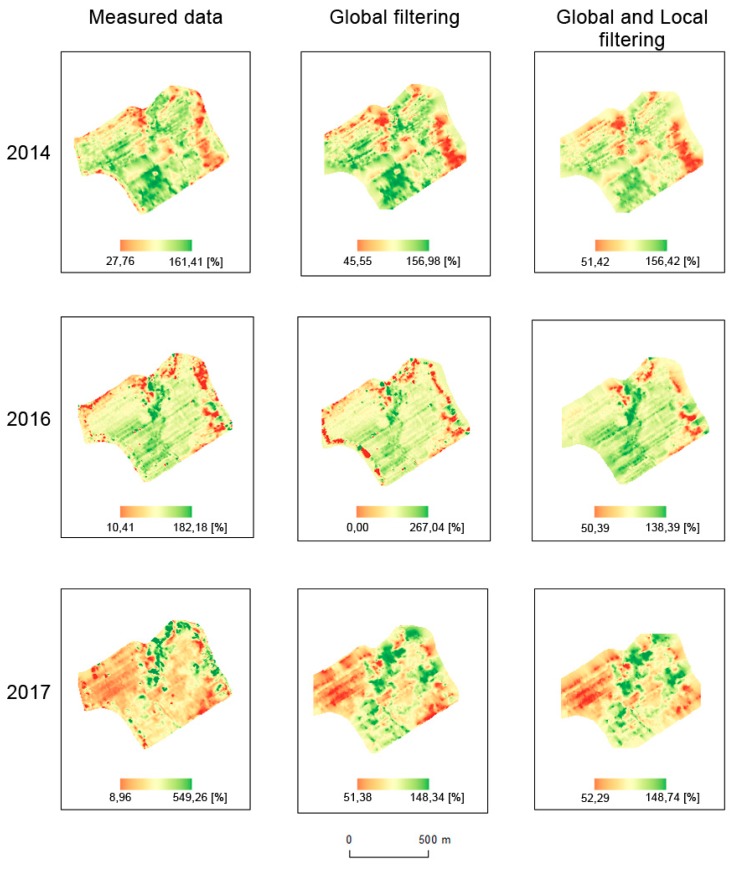
Interpolations of measured, globally filtered, and both globally and locally filtered data for the Pivovárka field (%).

Interpolations were made for all datasets (see Figure 12 and Figure 13). Simple Kriging was used as an interpolation method, as mentioned in Section 2.4; the parameters are presented in Table 4 and Table 5. For filtered data, spatial extents were smaller due to global filtration. In order to achieve homogeneous (consistent) and comparable results, we decided not to use extrapolation methods, as their precision in the respective areas would have been debatable. 

### 3.4. Verification of Sensor Measurements versus Filtered Data

The statistical analyses provided answers to the question of differences between interpolated surfaces. There were significant differences between the

- measured non-filtered data and measured data filtered by means of global filtering in six cases at a significance level of 95%;
- measured non-filtered data and measured data filtered by means of global and local filtering at a significance level of 95% in all cases.

**Figure 14:**
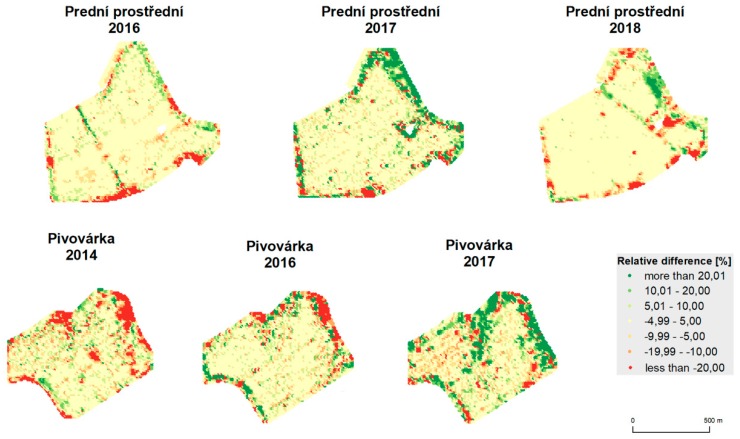
Relative difference between unfiltered data and data filtered by global filtering.

**Figure 15:**
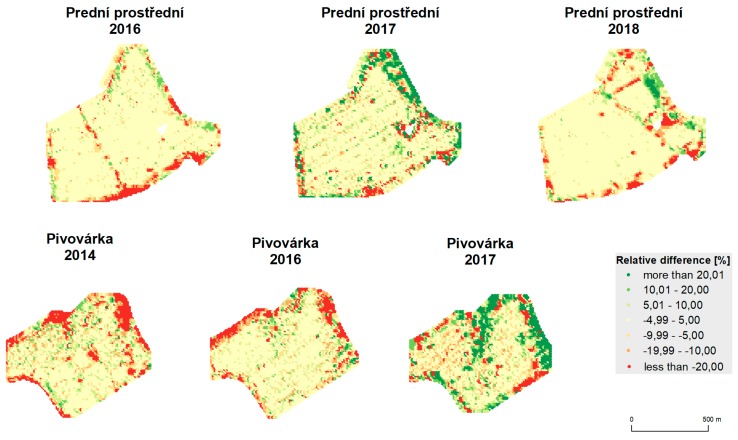
Relative difference between unfiltered data and data filtered by global and local filtering.

The results of statistical analyses are depicted in Table 6. Means, medians, standard deviations, testing criteria (t-score), and values (*p*) are provided for both fields and all years. Spatial relative differences, as depicted in Figure 14 and Figure 15, reveal the following spatial patterns:

- mean values appear inside the fields;
- deviations appear at the edges of the fields and around green infrastructure (groves);
- less commonly, deviations also appear inside the fields, mostly because of trajectory overlaps;
- influences of terrain and water flow accumulation characteristics appear in extreme climatic conditions (see Pivovárka field in 2017 as the most visible example).

Table 7 and Table 8 quantify the spatial distribution statistically. They depict the percentage of the area of the whole field that belongs to a relative difference category.

## 4. Discussion

The basic scientific questions raised at the beginning of the paper deal with the benefits of both global and local filters. We may formulate the following discussion points based on the achieved results. The presented discussion points are structured with the following logic. Point (a) compares the achieved results with results from related papers. Points (b) and (c) target usability issues of global and local filtering. Points (d), (e), and (f) summarize lessons learned from applications and verifications of filtering techniques.

**Figure 16:**
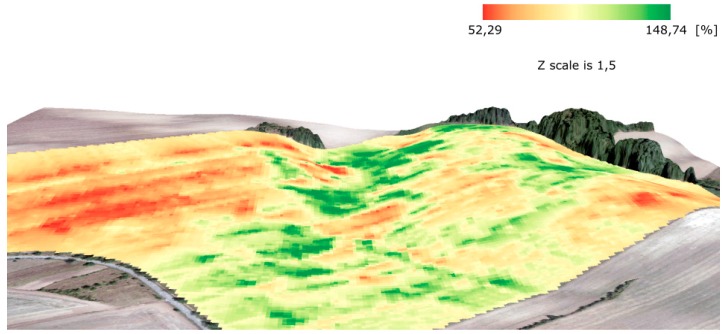
3D visualization of the measurements processed by means of global and local filtering for the Pivovárka field in 2017. Note the valley with a yield close to 150% of the average for the whole field (in green).

**Figure 17:**
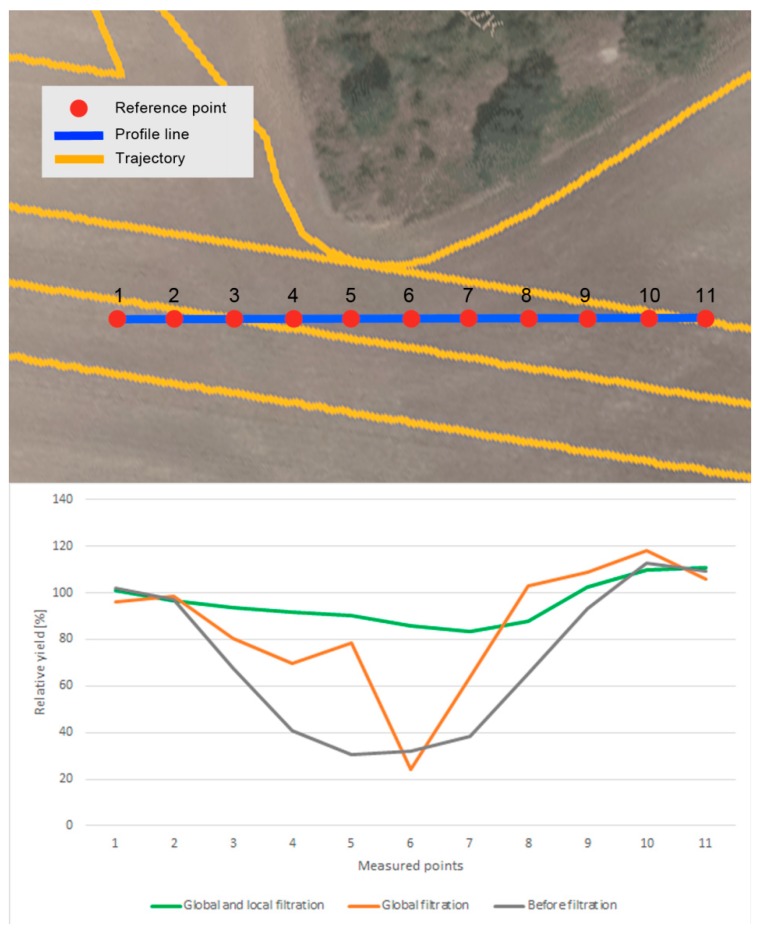
The differences in relative yield values for (1) interpolation from measured data, (2) interpolation from measured data that were processed by global filters, and (3) interpolation from measured data that were processed by global and local filters.

- The differences between (1) measured data, (2) measured data filtered globally, and (3) measured data filtered globally and locally were discovered as considerable in the analyzed fields and years. Similar results were achieved by Spekken, Anselmi, and Molin [17]. By comparing the global method (based on the removal of outliers from the histogram) and the local method proposed by Spekken, Anselmi, and Molin [17], it was similarly confirmed that local filtering has the effect of a low-pass filter as in other kinds of image processing domains (see e.g., Zhou et al. [27] for low-pass filtering).
- Global filtering represents a set of universal approaches that may be applied to any field. It was confirmed that global filtering is a must to eliminate high-level bias. The statement by Vega et al. [28] was also confirmed in our study, i.e., that global filtering improves yield distributions, while local filtering impacts the yield spatial structure for output interpolations and yield maps.
- The benefits of local filters were as follows:
  ○ The shape of a field: a convex shape lowers the need for local filtering. The more the shape of a field differs from a convex one, the higher the probability of crossed trajectories is, these causing bias in interpolations as well as in interpretations.
  ○ The variability of a relief: in particular, narrow valleys with steep slopes in a field require higher filtering efforts. Such areas, as depicted for instance in Figure 16, were characterized as areas with considerably higher yields than the average for the field. The conducted measurements showed that yields in such narrow valleys within a field may reach 150% of the average for the whole field or even higher. Agronomists at the Rostěnice Farm also confirmed that such very productive strips of land had existed there for the last two decades. It was stated that underground water from the slopes moves to the bedrock of the valley and, together with transported nutrients, creates a supernormal strip of fertile land. In such cases, a field harvester moves first along the bottom of the narrow valley and subsequently follows its “normal” trajectory, as planned for the whole field; see also Figure 16.
- Harvesting strategy: in general, the measurement functions differ when a field harvester goes from an area that is being harvested into an area that has already been harvested and/or is not intended for harvesting and vice versa; see Figure 17. When a field harvester goes from an area that is being harvested into an area that has already been harvested and/or is not intended for harvesting, the measured values fall to zero almost immediately. In contrast, when a field harvester goes from an area that has already been harvested and/or is not intended for harvesting into an area that is being harvested, the measurements are not continuous in the analyzed fields for an interval of between 5 and 30 m. In our study, this interval varied considerably while no universal influencing factor was revealed. As depicted in Figure 12 and Figure 13, the interpolation from measured data creates so called bull’s eyes (as also described by Wilson et al. [29]; Garnero et al. [30]). Such a phenomenon results in the prediction of very low values, i.e., close to zero, in the interpolated positions. In contrast, global as well as local filters have a smoothing function. A simple rule may be defined that local filters have a higher smoothing function than global filters, as clearly visible in Figure 17.
- This paper brought answers from the statistical point of view concerning differences among interpolations from filtered and non-filtered data. Applied sciences deal in this case, among others, with time and/or financial investments into different kinds of filtering. Three fields in Rostěnice farm, different from those used for statistical evaluations, were used to compute the time investments into various techniques presented in this paper. The results of time investments are presented in Table 9, broken down according to interpolations from (1) measured data that were globally filtered, (2) measured data that were locally filtered, and (3) measured data that were globally and locally filtered. Results presented in Table 9 can be summarized as follows:
  ○ Global filtering is a simple task, i.e., it required on average about three times less time than local filtering. The process of global filtering could potentially be more automatized than in this study, which would decrease the time investments even further.
  ○ Time investments into local filtering vary considerably among the fields. The number of measured points and the area of a field are the main drivers for time investments. Local filtering techniques also require local knowledge from the farm in order to apply a local filter accordingly. Time investments into local filtering shown in Table 9 are valid only for the cases in which a person performing the filtering and interpolations has local agronomical knowledge in the analyzed field. However, this was not our case, and therefore we needed two more hours on average for a field to discuss the filtering parameters with agronomists at the Rostěnice farm. In our case, the additional 2 h on average for a field were required to search for notes from the field, verify related evidence in the Farm Management Information System (FMIS), etc. It was confirmed that no general guidance for local filtering can be given.
  ○ Table 9 also depicts a “time index” that expresses the relative time investments in minutes per hectare.
- We may identify two main subjects for discussion from the spatial accuracy point of view:
  ○ In theory, the maximum positional error for the conducted measurements could be up to 18.6 m, though, in practice, such a value would be improbable. The positional error is influenced by two factors. The first is the speed of the harvester—in our study, this was 1.55 m·s^−1^, which was recommended as optimal at the Rostěnice Farm for cereal harvesting with a CASE IH AXIAL FLOW 9120 field harvester equipped with an AFS Pro 700 monitoring unit. The second is the delay between the collection of grain and the computation of the respective yield—in our study, a period of 12 s. The CASE IH AXIAL FLOW 9120 field harvester equipped with an AFS Pro 700 monitoring unit automatically links the computed yield value to the corresponding position, i.e., the position in which the field harvester was 12 s before. A positional error may arise when the speed of the field harvester is considerably higher or lower than the recommended one, meaning that the yield is not computed correctly for the corresponding position.
  ○ The positional error caused by the GNSS-RTK receiver was in centimeters. This means that the bias in measured positions resulting from the GNSS-RTK measurements could be considered negligible when considering the changing speed of the field harvester.

## 5. Conclusions

The presented paper in a novel way statistically and spatially evaluates the extent between non-filtered and filtered data measured by field harvesters. Similarly, from the originality point of view, time investments into filtering techniques were expressed as well. Among others, “time index” that expresses the relative time investments was provided as well for transferability and comparability of results.

The conducted yield measurements and subsequent data processing were performed to test the scientific questions raised at the beginning of the paper:

1. At a confidence level of 95%, there is a significant difference between interpolations from data measured by a field harvester and interpolations from the identical measured data processed by global filters.
2. At a confidence level of 95%, there is a significant difference between interpolations from data measured by a field harvester and interpolations from the identical measured data processed by global and local filters.

Both scientific questions were answered with respect to measurements of cereals in two fully operational Czech fields. The measured data may be influenced by a bias due to calibration. This paper addressed such a bottleneck by means of the relative comparison of measured data, and global and local filtering instead of using absolute measured values. The bias in the measured data did not have a considerable influence on the comparison of the resulting rasters.

It was confirmed that global filters are a must for any interpolations and/or interpretations. Local filters, on the other hand, lose their importance when all of the following conditions are met:

1. the shape of the analyzed field is convex;
2. the analyzed field is flat from a vertical perspective;
3. the recommended speed of harvesting is constant and follows the recommendations for the performing field harvester;
4. there are no barriers within a field, such as
  - green infrastructure like forest islands or wetlands,
  - technical infrastructure comprising poles for electrical cables, water infrastructure for ameliorations etc.,
  - geodetical and other signs,
  - other kinds of barriers such as stones, because of which the harvesting height above the ground needs to be adjusted;
5. crops, in our case cereals, are not damaged due to hailstorm and/or any other similar phenomena;
6. trajectories of a field harvester are not crossed.

Local filters become valuable when any combination of the above-mentioned conditions are not met. In general, local filters are, from a spatial perspective, often used for headlands and buffers around barriers. The topic of local filtering goes beyond the scope of this paper due to its complexity as well as need of detailed local knowledge from farm agronomists.

The presented six conditions are also important for algorithms that aim to set the optimal trajectory of farm machinery. As far as the authors know, no algorithm has been developed that would take into account the complexity of all six conditions. The further development of track-optimizing algorithms is needed because of the use of autonomous and, more commonly, semi-autonomous farm machinery under the Controlled Traffic Farming approach. Semi-autonomous driving has become a common feature of farm machinery as it automatically enables such machinery to follow defined trajectories in lines, with human-driver input required only for the negotiation of headlands and/or barriers.

The conducted research is also a resource for further research that will attempt to compare the measured yield with that predicted from yield productivity zones. In the cases of both measurement and prediction, such yield productivity zones are interpolated surfaces. The resulting interpolated surfaces are significantly influenced by the applied filtering techniques. Filtering therefore influences the evaluation of yield productivity predictions that are confronted with measured values.

## Figures and Tables

**Table 1:** Absolute number of sensor measurements, relative density of sensor measurements per hectare, and crops for each year and field.

Name	Date of Harvest	Crop	Number of Measurements	Density per Hectare
Přední prostřední	24 October, 2016	Wheat Winter	16,587	271.0
	14 July, 2017	Barley Spring	25,580	418.0
	7 July, 2018	Barley Spring	19,381	316.7
Pivovárka	11 October, 2014	Corn	20,232	454.7
	24 July, 2016	Barley Spring	34,710	780.2
	14 July, 2017	Barley Spring	16,038	360.4

**Table 2:** Absolute values of measurements filtered by means of global and local filtering for the Přední prostřední field.

Year	2016			2017			2018		
Filtration	Measured Data	Global Filtering	Global and Local Filtering	Measured Data	Global Filtering	Global and Local Filtering	Measured Data	Global Filtering	Global and Local Filtering
Number of points	16,587	11,223	10,542	25,580	21,158	20,968	19,381	13,800	13,612

**Table 3:** Absolute values of measurements filtered by means of global and local filtering for the Pivovárka field.

Year	2014			2016			2017		
Filtration	Measured Data	Global Filtering	Global and Local Filtering	Measured Data	Global Filtering	Global and LOCAL Filtering	Measured Data	Global Filtering	Global and Local Filtering
Number of points	20,232	15,527	14,663	34,710	29,785	28,950	16,038	12,485	11,404

**Table 4:** Exploratory Spatial Data Analysis (ESDA) results for setting up the Simple Kriging interpolation for the Přední prostřední field. Parameters were computed separately for (1) interpolation from measured data, (2) interpolation from measured data that were processed by global filters, and (3) interpolation from measured data that were processed by global and local filters.

Year	2016			2017			2018		
Filtration	Measured Data	Global	Global and Local	Measured Data	Global	Global and Local	Measured Data	Global	Global and Local
Lag Size	4.668	2.243	6.520	3.285	4.048	18.516	13.054	12.157	14.794
Number of Lags	12	12	12	12	12	12	12	12	12
Root-Mean-Square Standardized	1.169	1.823	0.827	10.853	12.630	0.977	1.413	0.963	0.895
Average Standard Error	6.233	3.630	7.134	1.229	0.707	8.690	4.974	6.900	7.009
Root-Mean-Square	5.559	5.359	5.306	9.324	8.567	8.399	6.885	6.505	6.291

**Table 5:** ESDA results for setting up the Simple Kriging interpolation for the Pivovárka field. Parameters were computed separately for (1) interpolation from measured data, (2) interpolation from measured data that were processed by global filters, and (3) interpolation from measured data that were processed by global and local filters.

Year	2014			2016			2017		
Filtration	Measured data	Global	Global and Local	Measured data	Global	Global and Local	Measured data	Global	Global and Local
Lag Size	16.000	13.922	13.433	5.234	4.449	25.560	4.233	22.468	21.469
Number of Lags	12	12	12	12	12	12	12	12	12
Root-Mean-Square Standardized	0.755	0.901	0.920	10.361	10.516	0.919	7.313	0.621	0.616
Average Standard Error	9.468	7.593	7.729	0.616	0.630	6.800	1.286	12.604	12.496
Root-Mean-Square	6.316	5.985	5.968	5.590	5.510	5.446	9.239	7.837	7.666

**Table 6:** Descriptive statistics and results of dependent *t*-test for paired samples. As the number of comparison points was large and the mean and median values were different by less than 10% in all cases, we expect a normal distribution.

Field	Year	N	Measured Data			Global Filtering			Global and Local Filtering			Measured Data Versus Global Filtering		Measured Data Versus Global and Local Filtering	
			m	med	stdv	m	med	stdv	m	med	stdv	t	*p*-val.	t	*p*-val.
Přední prostřední	2016	24,767	101.514	101.957	12.164	102.859	102.404	10.014	103.914	103.745	7.792	−25.7548	0.0000	-45.0429	0.0000
	2017	23,735	101.844	96.853	37.355	97.915	96.419	25.197	98.034	96.514	15.668	18.0595	0.0000	18.1743	0.0000
	2018	24,292	98.987	99.498	16.400	99.209	99.881	13.078	99.907	100.274	12.504	−3.9961	0.0001	−15.6783	0.0000
Pivovárka	2014	17,842	101.169	105.895	26.215	103.790	106.831	21.886	105.192	107.592	21.005	−29.3886	0.0000	−40.4324	0.0000
	2016	18,040	98.039	101.060	18.387	97.389	101.452	25.626	100.478	101.553	13.757	4.0418	0.0001	−30.0504	0.0000
	2017	17,524	103.458	95.371	43.107	94.981	93.605	21.239	94.865	92.773	19.882	34.4959	0.0000	33.3951	0.0000

**Table 7:** Relative difference between measured data and globally filtered data, and measured data and both globally and locally filtered data for the Přední prostřední field (%).

Relative Difference	Percentage of an Area Belonging to a Relative Difference Category					
	2016		2017		2018	
	Global Filtering	Global and Local Filtering	Global Filtering	Global and Local Filtering	Global Filtering	Global and Local Filtering
<−20%	4.04	5.73	4.77	5.81	4.21	5.70
−20%–−10%	3.29	3.72	2.95	4.91	4.08	5.04
−10%–−5%	8.81	6.48	7.93	8.18	4.62	6.01
−5%–5%	73.28	77.17	61.97	65.97	77.38	79.15
5%–10%	6.59	4.57	8.33	8.70	4.21	4.78
10%–20%	3.38	2.28	5.25	3.94	3.90	3.51
>20%	0.59	0.00	8.75	8.29	1.60	1.51

**Table 8:** Relative difference between measured data and globally filtered data, and measured data and both globally and locally filtered data for field Pivovárka (%).

Relative Difference	Percentage of an Area Belonging to a Relative Difference Category					
	2014		2016		2017	
	Global Filtering	Global and Local Filtering	Global Filtering	Global and Local Filtering	Global Filtering	Global and Local Filtering
<−20%	10.85	14.41	6.93	9.74	6.19	8.43
−20%–−10%	8.40	10.02	5.35	7.72	6.55	7.37
−10%–−5%	10.83	12.51	7.20	9.94	1.56	13.34
−5%–5%	52.58	58.97	60.86	67.06	43.74	44.55
5%–10%	11.85	12.41	9.30	10.06	9.30	10.67
10%–20%	5.12	5.32	5.15	3.90	8.57	9.30
>20%	1.36	0.77	5.21	1.32	13.45	14.76

**Table 9:** Time investments for interpolations from (1) measured data that were globally filtered, (2) measured data that were locally filtered, and (3) measured data that were globally and locally filtered.

Testing Field	Number of Measured Points	Area (ha)	Global Filtering		Local Filtering		Global and Local Filtering	
			Approximate Time consumption (min)	Time Index (min/ha)	Approximate Time Consumption (min)	Time Index (min/ha)	Approximate Time Consumption (min)	Time Index (min/ha)
Zákostelní	28,658	87.02	9.25	0.11	36.5	0.42	45.75	0.53
Miletovsko	8,406	42.52	7.75	0.18	16.5	0.39	24.25	0.57
Koberska	18,462	80.08	8.00	0.09	25.0	0.31	33.00	0.41
**Average**	**18,509**	**69.87**	**8.50**	**0.13**	**26.0**	**0.37**	**34.33**	**0.50**

## Supplementary Materials

The following are available online at https://www.mdpi.com/1424-8220/19/22/4879/s1, Figure S1: Fields managed by Rostěnice farm and hypsometry; Figure S2: Fields managed by Rostěnice farm and actual land cover.
