# Supplementary material for: Deployment and Verifications of the Spatial Filtering of Data Measured by Field Harvesters and Methods of Their Interpolation: Czech Cereal Fields between 2014 and 2018
Source: Sensors (Basel). 2019 Nov 8;19(22):4879. doi: 10.3390/s19224879 (PMC6891319; doi:10.3390/s19224879)
Supplement: Supplementary file 1 [file sensors-19-04879-s001.pdf]

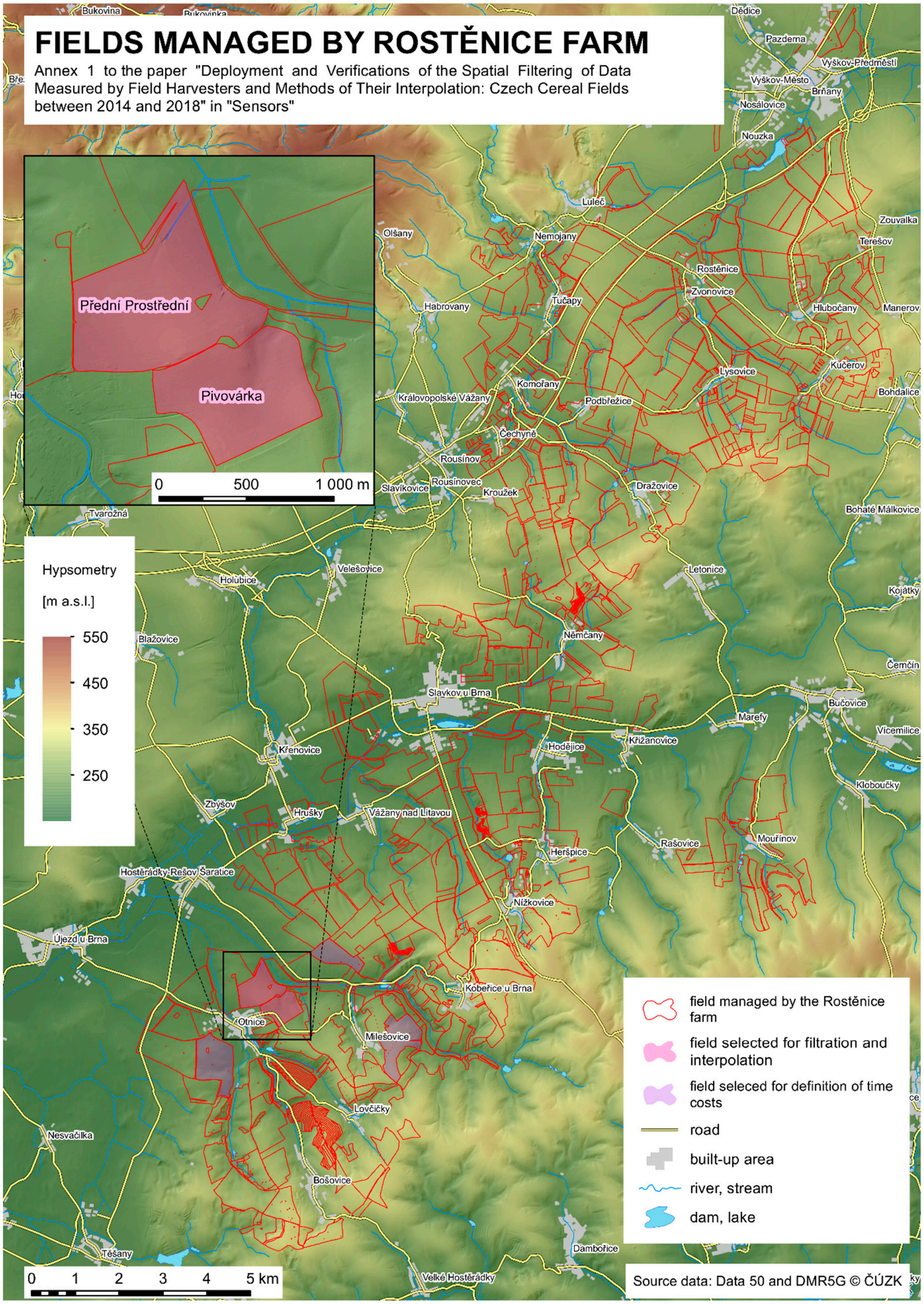

Figure S1. Fields managed by Rostěnice farm and hypsometry.

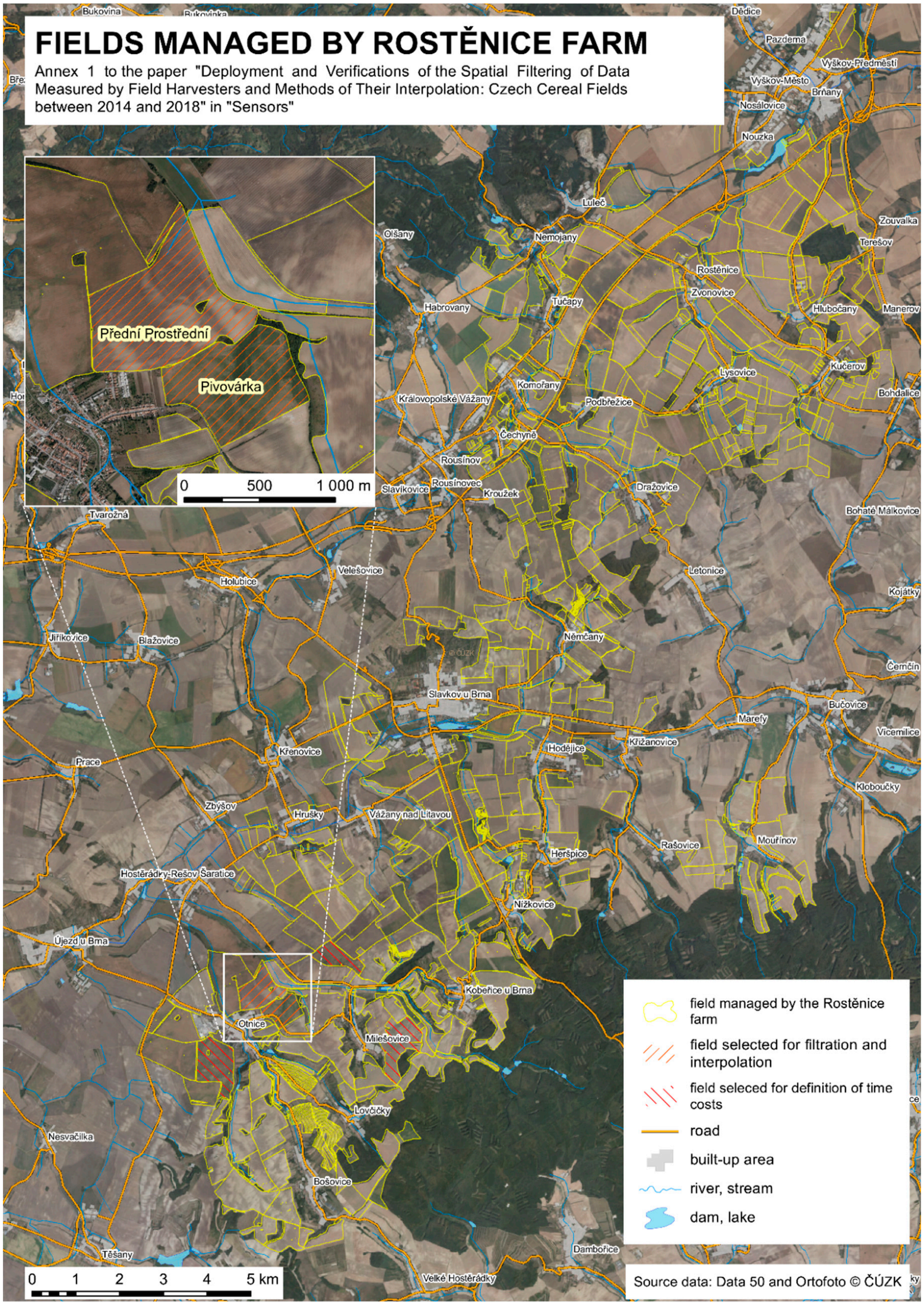

Figure S2. Fields managed by Rostěnice farm and actual land cover.
